# Molecular marker dissection of stem rust resistance in Nebraska bread wheat germplasm

**DOI:** 10.1038/s41598-019-47986-9

**Published:** 2019-08-12

**Authors:** Amira M. I. Mourad, Ahmed Sallam, Vikas Belamkar, Stephen Wegulo, Guihua Bai, Ezzat Mahdy, Bahy Bakheit, Atif Abo El-Wafa, Yue Jin, P. Stephen Baenziger

**Affiliations:** 1 Department of Agronomy and Horticulture, Plant Science Hall, UNL, Lincoln, NE, USA; 2 Department of Plant Pathology, Plant Science Hall, UNL, Lincoln, NE, USA; 3 USDA-ARS Hard Winter Wheat Genetics Research Unit, 4008 Throckmorton Hall, Manhattan, KS USA; 4 Agronomy Department, Faculty of Agriculture, Assiut University, Assiut, Egypt; 5 Department of Genetics, Faculty of Agriculture, Assiut University, Assiut, Egypt; 6 USDA-ARS Cereal Disease Lab, St. Paul, MN USA

**Keywords:** Agricultural genetics, Plant breeding

## Abstract

Stem rust (caused by *Puccinia graminis* f. sp. *tritici*) is a major disease of wheat. To understand the genetic basis of stem rust resistance in Nebraska winter wheat, a set of 330 genotypes representing two nurseries (DUP2015 and TRP2015) were evaluated for resistance to a Nebraska stem rust race (QFCSC) in two replications. The TRP2015 nursery was also evaluated for its resistance to an additional 13 stem rust races. The analysis of variance revealed significant variation among genotypes in both populations for stem rust resistance. Nine stem rust genes, *Sr6*, *Sr31*, *Sr1RS*^*Amigo*^, *Sr*2*4*, *Sr36*, *SrTmp*, *Sr7b*, *Sr9b*, and *Sr38*, were expected and genotyped using gene-specific markers. The results of genetic analysis confirmed the presence of seven stem rust resistance genes. One genotype (NE15680) contained targ*et* alleles for five stem rust resistance genes and had a high level of stem rust resistance against different races. Single marker analysis indicated that *Sr24* and *Sr38* were highly significantly associated with stem rust resistance in the DUP2015 and TRP2015 nurseries, respectively. Linkage disequilibrium analysis identified the presence of 17 SNPs in high linkage with the *Sr38*-specific marker. These SNPs potentially tagging the *Sr38* gene could be used in marker-assisted selection after validating them in additional genetic backgrounds.

## Introduction

Wheat (*Triticum aestivum* L.) is the third most widely produced crop in the U.S. following corn (*Zea mays* L.) and soybeans (*Glycine max* (L.) Merr). During the wheat life cycle, it is exposed to biotic and abiotic stresses that reduce grain yield and quality. One of these biotic stresses is stem rust disease (caused by *Puccinia graminis* f. sp. *tritici* Erikss. & E. Henning). In the past, stem rust was a major disease and caused significant yield losses^1,2^. Currently, in the central U.S. Great Plains, stem rust occurs rarely due to the effective incorporation of genetic resistance in breeding programs, such as the collaborative USDA-University of Nebraska Wheat Improvement Program. For the past 70 years, this program has aimed to release new cultivars that meet market needs of not only grain yield and quality, but also stem rust resistance. In this program, crosses between genotypes containing target traits are followed by phenotypic and genotypic selection for improved genotypes^3^. The selection is based upon four criteria: 1. winter survival, 2. stem rust resistance, 3. agronomic performance, and 4. end-use quality. Understanding the genetic basis of stem rust resistance will make it possible to more knowledgeably select genotypes effective against different races.

Generally, there are two types of stem rust resistance used in plant breeding programs: all-stage resistance (ASR) and adult plant resistance (APR). The main difference between the two types is that ASR produces a high level of resistance in all stages of the plant development including seedling resistance which allows easy selection. Adult plant resistance is, however, effective only beginning at the boot stage^4^. All-stage resistance is also known for “boom and bust cycles” due to the probability of a resistance gene when deployed singly being defeated by new stem rust races. Using a single major gene for stem rust resistance in wheat generally was found not to be durable for resistance due to the frequent emergence of new virulent *P*. *graminis* races^5^. To increase durability, gene pyramiding has been suggested as a highly effective strategy^6^.

The first step in pyramiding stem rust resistance genes is to identify the different resistance genes in elite genotypes that may be used as parents to create new pyramids in new cultivars^7^. The gene-for-gene theory is very helpful to postulate the presence of seedling genes. Genes can be postulated based on the infection type (ITs) of the different stem rust resistance genes to known stem rust races. To confirm the presence of the postulated genes in the tested nurseries, DNA markers tightly linked to those genes could be used.

Marker-assisted selection (MAS) provides many benefits in plant breeding^8,9^. One of the most important benefits is that markers are highly heritable and can be screened at the seedling stage. Thus, plant breeders can select resistant plants without phenotypic screening. In addition, MAS is very helpful in identifying the tagged resistance genes which have been pyramided in one genotype^10^.

The objectives of this study were to (1) screen a set of 330 Nebraska winter wheat genotypes for resistance to stem rust, (2) identify the possible genes and gene pyramids in the elite genotypes, and (3) select genotypes with pyramided stem rust resistance genes to be used in breeding programs.

## Materials and Methods

### Plant materials

Two different nurseries were used in this study: DUP2015 nursery which consists of 270 diverse genotypes and TRP2015 nursery which contains 60 diverse genotypes. The structure of each nursery was previously described^11,12^. Basically, lines in the DUP (preliminary yield trial) are advanced to the TRP (advanced yield trial) the following year based upon agronomic performance, disease resistance, and end-use quality. Briefly, both nurseries are part of the Nebraska Wheat Improvement Program where the selection is routinely done using stem rust resistance as one of the criteria.

### Stem rust inoculation and screening

The DUP2015 nursery (270 genotypes) was evaluated for the predominant race in North America, QFCSC, in Nebraska in two replications (one at Lincoln, NE and one at USDA-ARS, at Manhattan, KS). The TRP2015 nursery (60 genotypes) was evaluated for resistance to races QFCSC, QTHJC, MCCFC, RCRSC, RKRQC, TPMKC, TTTTF, GFMNC, QCCSM and TTKSK (commonly known as Ug99) at the USDA Cereal Disease Laboratory at St. Paul, MN in one replication. Because race TTKSK is one of the races in the Ug99 race group, the resistant or heterogenous genotypes to this race were selected and evaluated again using three additional variants in the Ug99 race group; TTKST (with added virulence to *Sr24*), TTTSK (with added virulence to *Sr36*), and TTKTT (with added virulence to *Sr24* and *SrTmp*)^13^, as well as non-Ug99 races TKTTF and TRTTF that possess significant virulence combinations^14,15^. Testing with the Ug99 races was done to determine if there are resistant lines should those races be found in Nebraska. In addition, the TRP2015 nursery was evaluated against race QFCSC in a second replication at Lincoln, NE. This approach of screening the DUP2015 to the predominant race of stem rust in North America and then to multiple races in the TRP2015 was to save labor and resources. In the DUP2015, our goal was to make sure lines have stem rust resistance. In the TRP2015, there are fewer lines, so it is easier to test with multiple races of stem rust. All the evaluations were performed at the seedling stage using the method of Jin and Singh^16^ with some modifications as described in Mourad *et al*.^11^. Rust reactions were scored using a 0–4 infection type scale developed by Stakman *et al*.^17^. Genotypes with scores of 0 to 2 were considered as resistant, and 3 to 4 as susceptible.

In order to identify possible genes controlling the resistance in the tested populations, gene postulation was performed. The TRP2015 population was postulated based on the IT of eleven different stem rust races, avirulence/virulence formula for these races and pedigree information. The phenotypic data of the DUP2015 population was compared to the IT of the different stem rust genes at the seedling stage^18^. Pedigree information of this population was used to confirm the possible presence of the postulated genes.

### Statistical analysis of stem rust resistance

The 0–4 scale^17^ was converted to a linear scale of 0 to 9^7,11,19^ for statistical analysis. Heterogeneous genotypes that had resistant plants were grouped with the resistant lines because resistant lines could be selected from them. The analysis of variance for stem rust resistance values was done using R software^20^.

### DNA extraction and polymerase chain reaction (PCR) conditions

DNA was extracted for genotyping-by-sequencing (GBS), simple sequence-repeat (SSR), or sequence-tagged site (STS) markers that link to known rust resistance genes^11^. Based on the pedigrees of the tested genotypes, some stem rust resistance genes were expected in some genotypes and SSR and STS markers for these genes were screened for those genotypes. Genotyping for *Sr24*, *Sr38*, *Sr31*, and *Sr1RS*^*Amigo*^ was done at the USDA-ARS lab, Manhattan, KS. Polymerase chain reaction (PCR) amplifications for SSR and STS markers for these genes were performed in a Tetrad Peltier DNA Engine (Bio-Rad Lab, Hercules, CA, USA) with a 12 µl PCR mixture containing 1.2 µl 10x PCR buffer (Bioline, Taunton, MA, USA), 2.5 mM MgCl_2_, 200 µM of each dNTP, 50 nM forward tailed primer, 250 nM reverse primer, 200 nM M13 fluorescent-dye-labeled primer, 0.6 U *Taq* DNA polymerase, and about 60 ng template DNA. A touchdown PCR program was used for PCR amplification. Briefly, the reaction was incubated at 95 °C for 5 min then continued for 5 cycles of 1 min at 96 °C, 5 min at 68 °C with a decrease of 2 °C in each subsequent cycle, and 1 min at 72 °C. For another 5 cycles, the annealing temperature started at 58 °C for 2 min with a decrease of 2 °C for each subsequent cycle. Reactions then went through an additional 25 cycles of 1 min at 96 °C, 1 min at 50 °C, and 1 min at 72 °C with a final extension at 72 °C for 5 min.

For the remaining expected genes (*SrTmp* and *Sr36*), genotyping was done at the University of Nebraska-Lincoln. Polymerase chain reaction (PCR) for the available SSR markers (Supplementary Table 1) was performed in 15 μl volume. Each reaction consisted of 2 μl 20 ng DNA template, 3 μl GoTaq Flexi buffer (without MgCl_2_), 0.3 μl 0.25 mM dNTPs, 1.20 μl 25 mM MgCl_2_, 0.2 μl from 0.5 u/μl GoTaq Flexi Taq polymerase (Promega, Madison, Wisconsin, USA) and 0.75 μl 10 pmol of each primer. Superfine resolution (SFR) 3% Agarose gel was used to separate the SSR marker products. The differential line of each gene was included in the SSR genotyping to identify the target band size for each primer (Supplementary Table 2).

### Gel scoring and statistical analysis

The amplified products of SSRs were scored using a visual score, ABI PRISM 3730 DNA Analyzer (Applied Biosystems, Foster City, CA, USA) and by using Gel Analyzer 2010 software (http://www.gelanalyzer.com/). Converted phenotypic data, (0–9 scale) and the genotypic data were used to perform single marker analysis (SMA). The analysis was done using SAS version 9^21^, following this model:

$${\rm{Y}}={\rm{\mu }}+{\rm{f}}\,({\rm{marker}})+\mathrm{error},$$

where Y is the trait value, µ is the nursery mean, and f (marker) is a function of the molecular marker^22^.

The phenotypic variation explained by each marker was calculated for the markers that showed a significant effect on rust resistance based on the SMA. Box plots showing stem rust scores for each of the alleles of the significant markers were created using R package ‘ggplot2’^23^. Genotypes having outlier values in each group were excluded and the SMA was redone in order to confirm the significant effect of each marker.

### Genome-wide association analysis (GWAS) and linkage disequilibrium (LD)

In the DUP2015 nursery, the association between markers (35,128 SNPs) and stem rust data from the tested genotypes (270 genotypes) was analyzed using TASSEL 5.0 software^24^ after removing heterozygous SNPs. Due to the presence of population structure^11^, two models were tested, mixed linear model + kinship (MLM + K) and mixed linear model + kinship + Q-matrix (MLM + K + Q-matrix). The marker-trait association was tested against Bonferroni corrections and false discovery rate (FDR) at a significance level of 5% in both models as well as compared with α = 0.05 significance. As many genes were expected to exist in these nurseries, we also looked for the association of minor genes controlling the resistance. This was accomplished using Settlement of MLM Under Progressively Exclusive Relationship (SUPER) method employed by GAPIT-R package^25^.

In the TRP2015 nursery, the number of genotypes was too low for GWAS analysis (60 genotypes). To identify SNP markers that are significantly associated with significant resistance genes, linkage disequilibrium (*r*^2^) among the SNPs located on the targeted chromosome and gene-specific SSR markers for those significant genes were calculated using TASSEL software^24^. Values of LD (*r*^2^) were visualized in a heatmap generated by ‘LDheatmap’ R package^26^. After detecting SNP markers which had high LD with the resistance-gene-specific marker, SMA between these SNPs and stem rust resistance was done to confirm the association between these SNPs and the resistance gene using SAS software^21^ based on the same SMA model described previously. The allele effect and targeted allele of each SNP were calculated using TASSEL^24^. Positive values indicated genes associated with the marker decreased stem rust resistance (increased susceptibility), while negative values indicated genes associated with the marker allele for increased resistance.

### Candidate genes and gene annotation

To further investigate the association of the significant SNPs and stem rust resistance, gene models underlying these SNPs were identified using the reference genome assembly (IWGSC REF Seq v.1.0). The functional annotation of the detected gene models was investigated using the same reference genome. The expression of gene models at different development stages was compared using a wheat expression database (http://www.wheat-expression.com/) to provide additional understanding of these results.

### Key message

Identifying genotypes carrying many stem rust resistance genes is helpful to produce a wide range of resistance. We identified five genotypes carrying four genes that could be used in breeding programs.

## Results and Discussion

### Genetic variation and pyramiding of stem rust resistance genes

**Figure 1:**
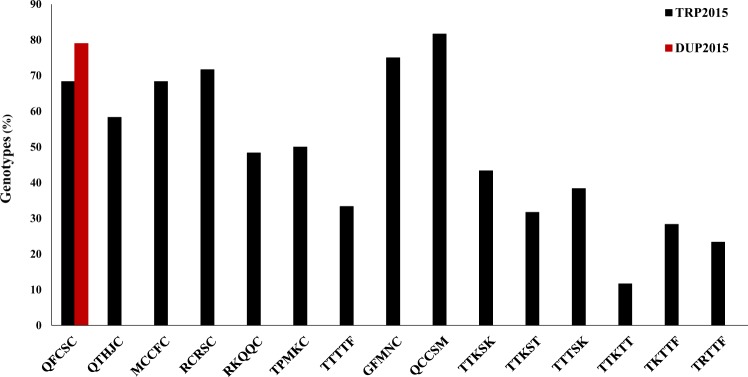
The percentage of genotypes showing resistance to different stem rust races. Red column represents the percentage of resistance genotypes in the DUP2015 nursery, while black columns represent the percentage of the resistance genotypes in the TRP2015 nursery.

The analysis of variance revealed highly significant differences among genotypes, but not between replications in both DUP2015 and TRP2015 nurseries^11^. Most of the DUP2015 nursery was resistant to race QFCSC with a percentage of 80% of the genotypes (Fig. 1), indicating the historic phenotypic screens and gene pyramiding for stem rust resistance in the breeding program was successful.

Many of the genotypes in the TRP2015 nursery were resistant to races QCCSM (82%), GFMNC (75%), RCRSC (72%), QFCSC (68%), MCCFC (68%), QTHJC (58%), TPMKC (50%), RKRQC (48%), TTKSK (43%), and TTTTF (33%) (Fig. 1). As TTKSK is one of the races in the Ug99 race group, we tested the 33 TTKSK-resistant genotypes against other races in the Ug99 race group, including TTTSK, TTKST, and TTKTT and found 23, 19 and seven genotypes were, respectively, resistant to these three races. Of the 39 genotypes that were evaluated using two non-Ug99 races, 17 and 14 genotypes were resistant to TKTTF and TRTTF, respectively. The high percentages of resistant genotypes to race QFCSC (80 and 68% in the DUP2015 and TRP2015, respectively) were expected in both nurseries due to routine evaluation and effective selection of advanced breeding lines for resistance to the different races in collaboration with USDA-ARS. As a result of this process, a high percentage of resistant genotypes was found in the TRP2015 nursery. The 14 genotypes which are resistant to TKTTF, TRTTF, and the Ug99 races could be used as a good source for breeding to stem rust resistance outside the U.S. as well as in the U.S. incase new races appear.

### Prediction and screening of stem rust resistance genes using molecular markers

The DUP2015 and TRP2015 nurseries were expected to contain many stem rust resistance genes such as *Sr24*, *Sr31*, *Sr6*, *Sr7b*, *Sr9b*, *Sr1RS*^*Amigo*^, *SrTmp*, *Sr36*, and *Sr38*. This expectation was determined based on the presence of these genes in the genetic background of the tested genotypes. For example, *SrTmp* was reported in “Goodstreak” in addition to *Sr6*^27–29^; *Sr7b* was reported in “Gage”^19^; *Sr36* and *Sr9b* were present in some northern USA cultivars^29,30^. The presence of these genes in the tested genotypes seems to be logical as those genes were widely used to improve wheat for stem rust resistance in the USA^31–33^. Two different SSR markers were used to screen the two nurseries for *Sr6* and the targ*et* alleles associated with this gene were found in many genotypes of those nurseries^11^. Moreover, *Sr6* was a major gene identified in the two populations [11], therefore, we focused on the other eight genes that were apparently less frequent in the current study.

To confirm the presence of the expected genes in the tested nurseries we compared the infection type (IT) of the genotypes with the widely accepted IT of each gene illustrated by McIntosh *et al*.^34^, which has been reported as a fast and effective approach to determine stem rust resistance genes^35^. However, this may not always give an accurate prediction of a gene due to the epistatic interaction between the different resistance genes. Therefore, it was necessary to use DNA markers to further investigate the presence of the expected resistant genes. Using molecular markers for specific stem rust resistance genes should be done side by side with gene postulation in order to confirm the presence of the resistance genes. For this purpose, the available specific SSR markers for each gene were used (Supplementary Table 1).

### Genetic analysis using gene-specific markers

As previously mentioned, nine stem rust genes were expected in both nurseries. *Sr6* gene was previously described^11^. Two expected genes (*Sr9b*, and *Sr7b*) were not included in this study due to the lack of good DNA markers for them. Markers for the remaining genes (*Sr24*, *Sr31*, *Sr1RS*^*Amigo*^, *SrTmp*, *Sr36*, and *Sr38*) were analyzed as follows.

#### *Sr36* gene

**Figure 2:**
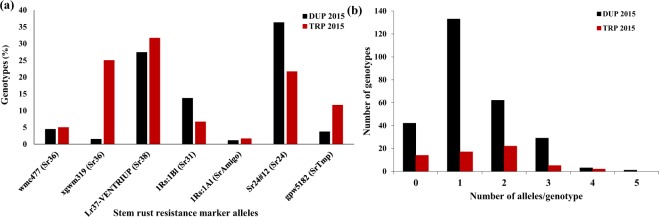
(**a**) percentage of genotypes having stem rust alleles in DUP and TRP populations and (**b**) Number of genotypes which having different stem rust alleles in both populations.

Among the four SSR markers used to predict the presence of the *Sr36* gene in the tested nurseries (Supplementary Table 1), only two markers (*Xwmc477* and *Xgwm319*) showed clear polymorphisms. Based on the positive control, ISr36, the target allele band size was 176 and 170 bp for *Xwmc477* and *Xgwm319*, respectively. In the DUP2015 nursery, 12 genotypes (4.4%) contained the target band of *Xwmc477* while four genotypes (1.5%) had the target allele of *Xgwm319*. In the TRP2015 nursery, 5.0 and 25% had the target alleles of *Xwmc477* and *Xgwm319*, respectively (Fig. 2a). Two biparental populations were previously tested for *Sr36* using the two SSR markers^34^. *X*w*mc477* co-segregated with *Sr36* in both populations while *Xgwm319* co-segregated with *Sr36* in one of the population and tightly linked to this gene at 0.9 cM distance in the other population^36^. In the current study, all the genotypes with *Xwmc477* target allele had IT of ;0 that conformed to the expected IT of *Sr36* gene. However, some of the genotypes with the target allele of *Xgwm319* showed ITs were not in accordance with expected *Sr36* IT (higher IT) (Supplementary Tables 3 and 4). *Xwmc477* appears to be a better marker than *Xgwm319* for detecting the presence of *Sr36*. However, by comparing the responses of the genotypes in the TRP2015 nursery to the different stem rust races, we found that some genotypes with *Sr36* marker alleles did not show the expected ITs for some races. For example, *Sr36* should exhibit IT of 0 or 0; to QFCSC and other Q races as well as to TTKSK and TTKTT. However, the genotypes carrying *Sr36* markers showed higher ITs to these races (Supplementary Table 5). Therefore, the discrepancy between *Sr36* marker data and phenotypic responses to different races remains to be elucidated. Such a discrepancy between the phenotypic response and marker data was found previously in some genotypes which confirms our conclusion^7^.

#### *Sr38* gene

The presence of *Sr38* was tested by the STS marker VENTRIUP-LN2^37^. The target allele band size of this STS marker is 259 bp in ISr38. In the DUP2015 nursery, 74 genotypes (27.4%) had the target band associated with the *Sr38* gene and 19 genotypes (31.7%) in the TRP2015 had the target band (Fig. 2a). All the genotypes with the *Sr38* gene had the expected IT for this gene (; to ;13 Supplementary Tables 3 and 4). In addition, the TRP2015 nursery showed IT data conforming the postulation for the presence of *Sr38* predicted by marker analysis, except for one genotype which showed highly susceptible responses to TMPKC and QTHJC races. Based on these results, we concluded that VENTRIUP-LN2 marker is a powerful marker in predicting the presence of this gene. *Sr38* is a very important gene as it is located on a translocation and tightly linked with *Lr37* resistance gene for resistance to leaf rust (caused by *P*. *triticina* Eriks) and *Yr17* for resistance to stripe rust (caused by *P*. *striiformis* Westend. f. sp. *tritici* Erikss)^4^. Those genotypes with *Sr38* gene can be used in further breeding for resistance to multiple rust diseases.

#### *Sr31* and *Sr1RS*^*Amigo*^ genes

SSR marker *Xstm120* was used to detect the presence of the rye chromosome arm (1RS) in the tested materials. 1RS contains important resistance genes to stem rust and powdery mildew (incited by *Blumeria graminis* D.C. f. sp. *tritici*)^34^ in addition to high yield traits^31,32^. In this study, 13.7% of the DUP2015 genotypes (37 genotypes) carry the 1BL.1RS translocation and contains *Sr31*. In the TRP2015 nursery, 6.7% of the genotypes (four genotypes) showed the presence of 1BL.1RS. Only a few genotypes had the 1AL.1RS translocation with *Sr1RS*^*Amigo*^ gene in both nurseries (three genotypes in the DUP2015 and one genotype in the TRP2015) (Fig. 2a). Most of the TRP2015 genotypes with the target band of *Xstm120* had ITs that agreed with the presence of *Sr31* and *Sr1RS*^*Amigo*^ for the different stem rust races, which confirms that this marker is a useful marker for detecting the presence of the two stem rust resistance genes.

#### *Sr24* gene

The STS marker Sr24#12 was used to predict the presence of the *Sr24* gene in both nurseries. This marker has been reported to be in a complete linkage with *Sr24* and hence it is effective in detecting the presence of this gene^38,39^. Based on the positive control isoline, ISr24, the target allele size of this marker is 500 bp. In the DUP2015 nursery, 36.4% of the genotypes (98 genotypes) contained the Sr24#12 target allele whereas in the TRP2015, 21.7% of the genotypes (13 genotypes) had the Sr24#12 target allele (Fig. 2a). Almost all the tested genotypes had ITs that were consistent with the presence of *Sr24* predicted by the marker (Supplementary Table 3 and 4). In addition, all the TRP2015 genotypes which had the target allele of the marker displayed the expected ITs for the presence of *Sr24* gene, except for two genotypes which showed a degree of susceptibility against some races (Supplementary Table 5). Based on our results and previous studies, we can conclude that the Sr24#12 STS marker is a useful marker for detecting the *Sr24* gene.

#### *SrTmp* gene

Three SSR markers were used to detect the presence of *SrTmp* gene in this study (Supplementary Table 1), but only marker *Xgpwm5182* showed a clear polymorphism. Based on the positive control isoline, ISrTmp, the target allele size of this marker is 174 bp. Using this marker, only 10 (3.7%) and seven (11.7%) genotypes were identified to carry the target band in the DUP2015 and TRP2015 nurseries, respectively (Fig. 2a). *Xgpwm5182* has been reported as a possible marker to detect the presence of *SrTmp* gene^40^. However, due to the large distance between the marker and *SrTmp* (1.8 cM) and the presence of another resistance gene in the same chromosomal position^41^, *Xgpwm5182* may not be predictive for *SrTmp* as was expected and more diagnostic markers are needed for this gene. Goodstreak in the TRP2015 nursery was expected to carry *SrTmp* in addition to *Sr6*^28,29^, however, it did not have the target band of this marker (Supplementary Table 4). In addtition, only two out of the seven genotypes with the tragetted marker band had ITs in agreement with the expected response to the different stem rust races. Therefore, this marker appears to be ineffective in predicting the presence of *SrTmp* gene. This contradiction confirms that this marker did not have any diagnostic value to detect this gene.

#### Single marker analysis and gene pyramiding

After screening gene-specific markers for all the expected genes in the tested nurseries, we found that the number of target alleles per genotype ranged from zero to five in the DUP2015 and from zero to four in the TRP2015, respectively (Fig. 2b and Supplementary Tables 3 and 4). Among the resistant genotypes identified phenotypically, 24 and eight genotypes did not have any target allele of the screened markers for the known resistance genes in the DUP2015 and TRP2015 suggesting that these genotypes might carry other stem rust resistance genes than those we screened with molecular markers. Based on the marker data, one, three, 29, and 62 genotypes contained five, four, three, and two stem rust resistance genes in the DUP2015 nursery; and two, five, and 22 genotypes contained four, three, and two stem rust resistance genes in the TRP2015 nursery. The presence of genotypes with more than one stem rust resistance gene was expected because the intermating between resistant genotypes has been a routine in the Nebraska Wheat Improvement Program. As this program has a long history in Nebraska, many genes have been inadvertently pyramided in its germplasm. Such genotypes with many stem rust resistance genes could be selected for excellent stem rust resistance, and potentially, both improved stripe rust and leaf rust resistance due to the existence of genes for resistance to multiple rusts including *Sr38*/*Lr37*/*Yr17*, *Sr24*/*Lr24*, and *Sr31*/*Lr26*/*Yr9*. Genotype NE15680 from the DUP2015 nursery and genotype NE14575 from the TRP2015 nursery are good examples of such genotypes (Supplementary Tables 3 and 4). Crossing between these two genotypes could be useful in developing wheat cultivars with at least five resistance genes (*Sr24*, *Sr31*, *Sr36*, *Sr6*, and *Sr38*) and hence more durable resistance to multiple stem rust races.

**Figure 3:**
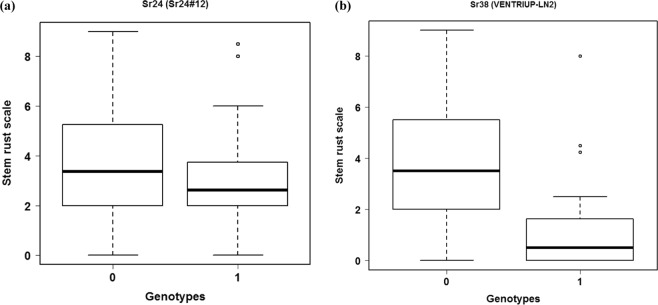
Box plot for the two significant genes in both populations: (**a**) the average of the individuals containing *Sr24 (denoted by 1)* in the DUP2015 population and the individuals which do not have this gene (denoted by 0). (**b**) The average of the individuals containing *Sr38 (denoted by 1)* in the TRP2015 population and the individuals which  do not have this gene (denoted by 0).

Due to the existence of many stem rust resistance genes in the germplasm of the tested nurseries, it is worth to identify genes with the major effect on the resistance using single marker analysis (Table 1). The analysis was done using the phenotyoic data of QFCSC race only as it is the most common race in Nebraska. In both nurseries, *Sr6* was highly significantly associated with the resistance^11^. In addition to this marker, Sr24#12 (*Sr24*) and VENTRIUP-LN2 (*Sr38*) had highly significant associations with stem rust resistance in the DUP2015 and TRP2015 nurseries, respectively with *p* values of 0.003 for both markers (Table 1). These two genes were the most frequent genes after *Sr6* in the DUP2015 (98 genotypes) and TRP2015 (19 genotypes). The phenotypic variation explained by marker (PVE) was calculated for the significant markers. Marker Sr24#12 (*Sr24*) had a PVE of 3% in the DUP2015 nursery, while, marker VENTRIUP-LN2 (*Sr38*) had a PVE of 15% in the TRP2015 nursery. For each significant gene, the phenotypic differences between the two genotypic groups with contrasting marker alleles (1 *vs* 0) in both nurseries is presented in Fig. 3. The average rust rating of the genotypes having *Sr38* gene in the TRP2015 nursery was in agreement with the linearized IT (“1”). The average rust rating of the genotypes having *Sr24* in the DUP2015 was lower than expected linearized IT (“4”). The average rust rating of genotypes without the *Sr24* and *Sr38* genes in both populations were also lower than expected IT for susceptible genotypes (5 or higher), indicating the presence of other resistance genes in the genotypes without *Sr24* and *Sr38*.

**Table 1:** Single marker analysis for stem rust resistance in the DUP2015 and TRP2015 nurseries.

	TRP2015	DUP2015
	*VENTRIUP*-*LN2*	*Sr24#12*
p value	0.003	0.003
PVE^a^	14.97%	3.25%
Allele frequency	0.32	0.36
Allele effects	1.07	0.46
Chromosome	2A	3D
Gene	Sr38	Sr24

^a^Phenotypic variation explained by marker.

### Association mapping for stem rust resistance

Structure analysis showed the presence of structure (k = 2) in the DUP2015 nursery^11^. Therefore, two models, MLM + k and MLM + k + Q-matrix were used in the GWAS. A set of 32 SNPs were previously found to be associated with the resistance on chromosome 2D and linked with *Sr6* gene^11^. Based on the SMA, *Sr24* (on chromosome 3D) was significantly associated with the resistance in the DUP2015 nursery. Therefore, it was worth to identify the significant SNPs associated with this gene using GWAS. However, the GWAS results did not reveal any significant SNPs located on that chromosome based on Bonferroni corrections 5%, FDR 5%, or at α = 0.05 significance level. The GWAS was performed also using SUPER software which increases the statistical power of the test^25,42^. However, the same result was obtained. The absence of significant SNPs could be explained by the lower PVE value (3.00%) of this gene in the DUP2015 nursery based on SMA (Table 1).

**Figure 4:**
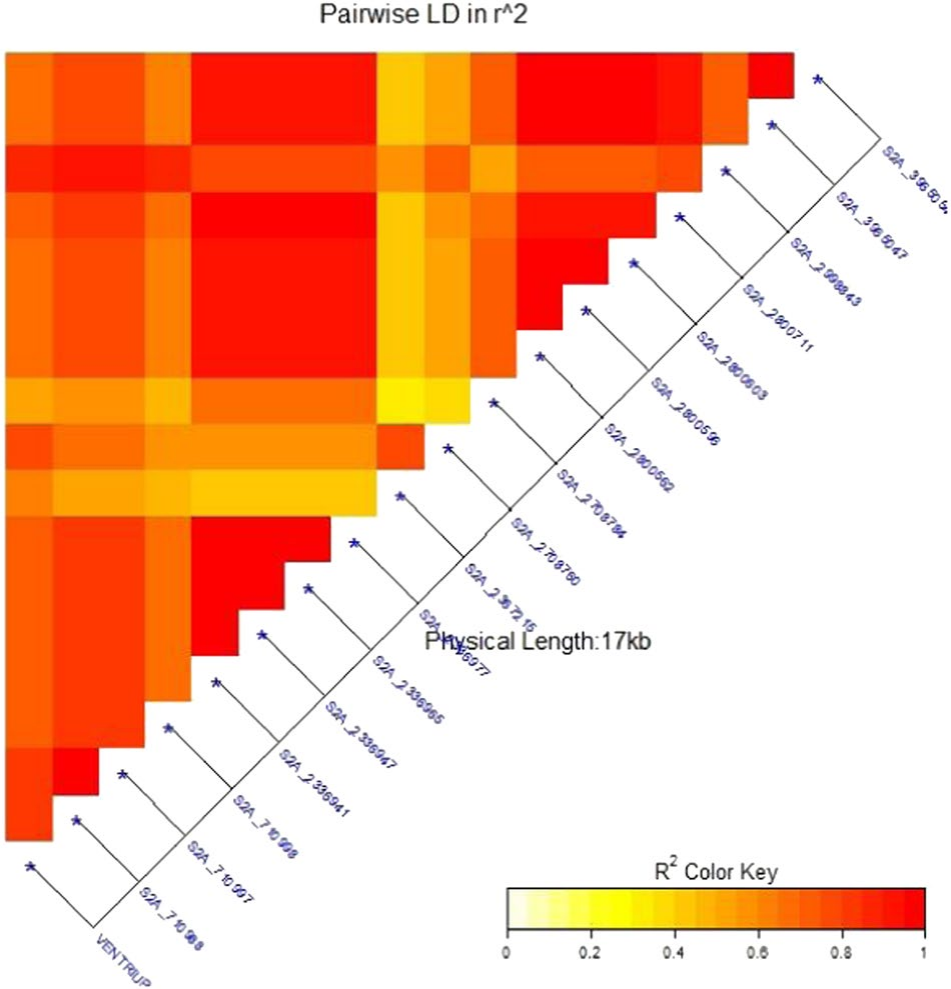
Linkage disequilibrium (LD) analysis in the TRP2015 population: heatmap of LD between the specific STS marker “VENTRIUP-LN2” for *Sr38* resistance gene and the 17 SNPs showed highly significant LD with the marker.

Because of the low number of the TRP2015’s genotypes (60 genotypes), GWAS was not applied as it was recommended to use a minimum of 100–500 individuals to detect a true marker-trait association^43^. Alternatively, in order to identify SNPs significantly associated with the resistance gene (*Sr38*), LD analysis was done between all SNPs located on chromosome 2A (1601 SNPs) and the specific STS marker, VENTRIUP-LN2 (Table 2 and Fig. 4). As a result, 17 SNPs were found to be linked to the STS marker with R^2^ values ranging from 0.71 for SNP S2A_2708784 to complete LD of 1.00 for SNP S2A_2367215 and S2A_2708760. The high LD between the STS marker and SNPs suggests that these SNPs would be excellent markers to predict the presence of this gene.

**Table 2:** Linkage disequilibrium (LD) between the specific *Sr38* marker (VENTRIUP-LN2) and SNPs located on chromosome 2A in the TRP2015. Single marker analysis between the significant SNPs and stem rust resistance. Gene models underlying them based on the IWGSCv1.0 and TGAC v1.0 data bases.

SNP_ID	*R* ^*2*^	*P*-value	F-value	P.V.E	Target allele^$^	Allele effect	Gene model (IWGSC v1.0)	Gene model (TGCA v1.0)
S2A_2367215	1.00	0.001	13.24**	30.62%	**C**:A	−2.18	TraesCS2A01G004100.1	TRIAE_CS42_2AS_TGACv1 _114644_AA0368550.1
S2A_2708760	1.00	0.003	10.70**	25.07%	**G**:A	−2.22	TraesCS2A01G005500.1	TRIAE_CS42_2AS_TGACv1 _113850_AA0361440.1
S2A_2708784	0.71	0.001	12.22**	19.96%	**G**:A	−3.23		
S2A_710988	0.91	0.002	10.26**	18.24%	**A**:G	−2.13	—	
S2A_710997	0.91	0.002	10.26**	18.24%	**C**:T	−2.13	—	
S2A_2336941	0.83	0.006	8.11**	13.50%	**T**:C	−2.25	TraesCS2A01G003700.1	TRIAE_CS42_2AS_TGACv1 _113891_AA0361970.1
S2A_2336947	0.83	0.006	8.11**	13.50%	**G**:C	−2.25		
S2A_2336965	0.83	0.006	8.11**	13.50%	**G**:A	−2.25		
S2A_2336977	0.83	0.006	8.11**	13.50%	**T**:C	−2.25		
S2A_2800711	0.83	0.008	7.69**	12.88%	**A**:G	−2.13	—	
S2A_2800562	0.82	0.003	10.43**	16.45%	**G**:A	−2.56	—	
S2A_2800596	0.82	0.003	10.04**	16.45%	**G**:A	−2.56	—	
S2A_2800603	0.82	0.003	10.04**	16.45%	**C**:G	−2.56	—	
S2A_2998843	0.82	0.018	6.03*	12.30%	**T**:A	−1.69	—	
S2A_3965047	0.75	0.008	7.52**	12.85%	**T**:C	−2.33	TraesCS2A01G010200.1	TRIAE_CS42_2AS_TGACv1_112938_AA0348130.2
S2A_3965054	0.75	0.008	7.52**	12.85%	**C**:T	−2.33		
S2A_710998	0.74	0.016	6.29*	12.02%	**C**:G	−1.64	—	

R^2^ Linkage Disequilibrium.

P.V.E. phenotypic variation explained by markers.

*P-value < 0.05.

**P-value < 0.01.

^**$**^left bold allele is associated with increasing stem rust resistance.

To confirm the association between these 17 SNPs and *Sr38* gene, SMA was done between these SNPs and stem rust resistance (Table 2). Among the 17 SNPs, 15 SNPs were highly associated with the resistance at a *p*-values ranging from 0.008 for SNPs S2A_2800711, S2A_3965047, and S2A_3965054 to 0.001 for SNPs S2A_2367215 and S2A_2708784. The remaining two SNPs (S2A_710998 and S2A_2998843) were significantly associated with the resistance at *p*-values 0.016 and 0.018, respectively. The phenotypic variation explained by those markers (PVE) were high and ranged from 12.02% for S2A_710998 to 30.62% for SNP S2A_2367215 (Table 2). The allele effect of each SNP marker was evaluated using stem rust linearized 0 to 9 score and it ranged from −1.64 for SNP S2A_710998. to −3.23 for SNP S2A_2708784. This result indicated that selecting for these significant SNPs could reduce the infection by more than 33%.

### Genes underlying significant SNPs and their functional annotations

To further understand the association between the significant SNPs associated with *Sr38* and the stem rust resistance in the TRP2015 nursery, genes containing these SNPs were annotated. Among the 17 SNPs, nine were located within four genes; TraesCS2A01G004100.1 (one SNP), TraesCS2A01G005500.1 (two SNPs), TraesCS2A01G003700.1 (four SNPs) and TraesCS2A01G010200.1 (two SNPs). The functional annotation was known for only three models of the identified models and the fourth one has unknown functional annotation (Table 3).

**Table 3:** Gene annotation of the four gene models underlying the significant SNPs and their probable function.

Gene model (IWGSC v1.0)	Gene annotation	Probable function	References
TraesCS2A01G004100.1	Disease resistance protein (TIR-NBS-LRR class) family	Disease resistance	https://www.ncbi.nlm.nih.gov/gene/834561
TraesCS2A01G005500.1	Late embryogenesis abundant (LEA)	Environmental stress resistance	^45^
TraesCS2A01G003700.1	Receptor like kinase	Plant defense	^46^
TraesCS2A01G010200.1	Unknown	—	—

**Figure 5:**
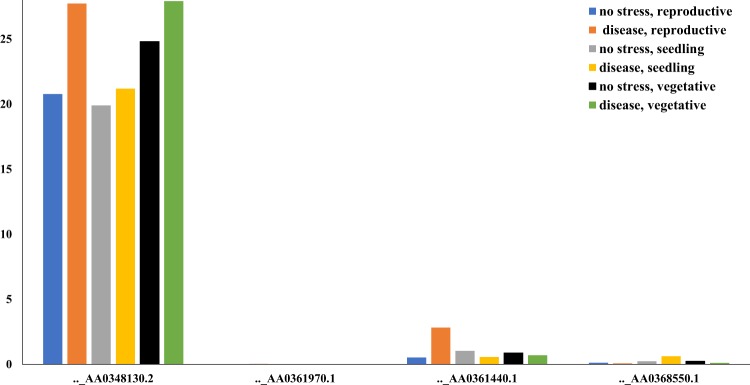
The expression of the gene models harboring SNPs significantly associated with stem rust resistance in the TRP2015 set. Blue, gray and black columns represent the gene expression under controlled conditions at reproductive, seedling and vegetative growth stages, respectively. While, orange, yellow and green columns represent the gene expression under disease infection conditions at the same growth stages.

The expression data of the four identified gene models under control and disease conditions was reported (Fig. 5). Two gene models have more expression under disease conditions compared with controlled conditions at the seedling stage (TRIAE_CS42_2AS_TGACv1_112938_AA0348130.2 = TraesCS2A01G010200.1 and TRIAE_CS42_2AS_TGACv1_114644_AA0368550.1 = TraesCS2A01G004100.1). The functional annotations of the first gene model was unknown. The second gene model (TRIAE_CS42_2AS_TGACv1_114644_AA0368550.1 = TraesCS2A01G004100.1) has a functional annotation associated with disease resistance as it produced TIR-NBS-LRR, one of the disease resistance protein families (Table 3). This protein family was reported to stay in the cell membrane and helps the plant to recognize the pathogen that attacks it. Some genes from this family are required for the defense activation of some resistance genes like Pto (bacterial resistance gene in tomato) and *Rpg1* (stem rust resistance gene in barley and rice)^44^. In our study, gene model TRIAE_CS42_2AS_TGACv1_114644_AA0368550.1 was underlying SNPs in a complete LD with the STS marker, had high P.V.E., large allele effect, and was highly significantly associated with the resistance (SNP_ID; S2A_2367215, S2A_2708760, and S2A_2708784). Based on these results, the identified SNPs could be converted to Kompetitive Allelic Specific PCR (KASP) markers and used for selecting the *Sr38* gene. However, more studies are required to further investigate the association between these SNPs and the *Sr38* gene. The LD analysis between SNPs and known specific DNA markers was very useful and could be an alternative way to identify candidate SNPs for specific genes.

In conclusion, the high percentage of resistant genotypes from the Nebraska Wheat Breeding Program provided evidence for the success of the program. In this program, many stem rust resistance genes have been incorporated and pyramided at various levels in some genotypes. Resistant genotypes carrying multiple stem rust genes could be selected and used in future breeding programs for resistance to a wide spectrum of pathotypes of stem rust, stripe rust, and leaf rust. SNP markers were not identified for the *Sr38* gene previously. The identified 17 SNPs using LD could be a reliable source for marker-assisted selection (MAS) for this gene. However, more studies should be done to confirm the association between these SNPs and the gene.

## Supplementary information

supplementary tables 1 (List of markers used to detect the presence of the different stem rust resistance genes, the expected band size, primer sequence and amplification conditions of each marker) and 2 (List of differential lines used as a positive control in marker-assisted selection to detect the right allele band size, the pedigree of each isoline and source as described on http://rusttracker.cimmyt.org/?page_id=30. ID was added to be used in the text.)

Supplemetary tables 3 Presence of the different marker alleles associated with the expected stem rust resistance gene in the DUP2015 genotypes),4(Presence of the different marker alleles associated with the expected stem rust resistance gene in the TRP2015 genotypes) and 5 (Response of the TRP2015 gnotypes to the different stem rust races, expected genes in each genotype based on the response to the different stem rust races, and the result of molecular markers. The evaluation was done based on Stakman scale (1962). For each genetic marker, "0" means the absence of the marker while "1" means the presence of the marker. Red cells indicate the disagreement between the genotypic and phenotypic data, while green cells represent the agrrement between both types of data.)

## References

[1] Leonard KJ, Szabo LJ (2005). Stem rust of small grains and grasses caused by Puccinia graminis. Mol. Plant Pathol..

[2] Eversmeyer MG, Kramer CL (2000). Epidemology of wheat leaf and stem rust in the central great plains of the USA. Annu. Rev. Phytopathol..

[3] Baenziger PS, Shelton DR, Shipman MJ, Graybosch RA (2001). Breeding for end-use quality: Reflections on the Nebraska experience. Euphytica.

[4] Roelfs, A. P., Singh, R. P. & Saari, E. E. Rust Diseases of Wheat: Concepts and methods of disease management. *Rust Diseases of Wheat*: *Concepts and methods of disease management*. (1992).

[5] Ellis JG, Lagudah ES, Spielmeyer W, Dodds PN (2014). The past, present and future of breeding rust resistant wheat. Front Plant Sci.

[6] Bajgain P (2015). Association mapping of North American spring wheat breeding germplasm reveals loci conferring resistance to Ug99 and other African stem rust races. BMC Plant Biol..

[7] Zhang D, Bowden RL, Yu J, Carver BF, Bai G (2014). Association analysis of stem rust resistance in U.S. winter wheat. PLoS One.

[8] Mohan Madan, Nair Suresh, Bhagwat A., Krishna T. G., Yano Masahiro, Bhatia C.R., Sasaki Takuji (1997). Molecular Breeding.

[9] Young ND (1996). QTL mapping and quantitative disease resistance in plants. Annu. Rev. Phytopathol..

[10] Anderson, J. A. Plant genomics and its impact on wheat breeding. in *Plant Molecular Breeding* (ed. Newbury) 184–215 (2003).

[11] Mourad AMI (2018). Genome-wide association study for identification and validation of novel SNP markers for Sr6 stem rust resistance gene in bread wheat. Front. Plant Sci..

[12] Belamkar V (2018). Genomic selection in preliminary yield trials in a winter wheat breeding program. G3 Genes|Genomes|Genetics.

[13] Newcomb M (2016). Kenyan isolates of Puccinia graminis f. sp. tritici from 2008 to 2014: Virulence to SrTmp in the Ug99 race group and implications for breeding programs. Phytopathology.

[14] Olivera PD (2012). Races of Puccinia graminis f. sp. tritici with combined virulence to Sr13 and Sr9e in a field stem rust screening nursery in Ethiopia. Plant Dis..

[15] Olivera P (2015). Phenotypic and genotypic characterization of race TKTTF of Puccinia graminis f. sp. tritici that caused a wheat stem rust epidemic in southern Ethiopia in 2013–14. Phytopathology.

[16] Jin Y (2007). Characterization of seedling infection types and adult plant infection responses of monogenic Sr gene lines to race TTKS of Puccinia graminis f. sp. tritici. Plant Dis..

[17] Stakman, E. C., Stewart, D. M. & Loegering, W. Q. *Identification of physiologic races of Puccinia graminis var*. *tritici*. *USDA*_*ARS* Washington, (USDA_ARS, 1962).

[18] Singh, S., Singh, Ravi, P. & Huerta-Espino, J. Stem rust. In *Disease Resistance in Wheat* (ed. Sharma, I.) 18–32 (CAB International 2012, 2012).

[19] Kumssa TT (2015). Characterization of stem rust resistance in wheat cultivar Gage. Crop Sci..

[20] Division, W. E. *Introduction to R Statistical Software*. **97333**.

[21] SAS Institute Inc. Base SAS® 9.4 Procedures Guide. *Statistical Procedures* Second Edi, Cary, NC: SAS Institute Inc. (2013).

[22] Francis, D. M., Merk, H. L. & Namuth-covert, D. Introduction to Single Marker Analysis (SMA). 1–3 Available at: http://www.extension.org/pages/32552/introduction-to-single-marker-analysis-sma (2011).

[23] Wickham, H. *ggplot2*: *Elegant Graphics for Data Analysis*. *Springer*-*Verlag New York*. (Springer Verlag, 2009).

[24] Bradbury PJ (2007). TASSEL: Software for association mapping of complex traits in diverse samples. Bioinformatics.

[25] Wang, Q., Tian, F., Pan, Y., Buckler, E. S. & Zhang, Z. A SUPER powerful method for genome wide association study. *PLos One* **9** (2014). 10.1371/journal.pone.0107684 PMC4172578 25247812

[26] Shin J-H, Blay S, McNeney B, Graham J (2006). LDheatmap: An R function for graphical display of pairwise linkage disequilibria between single nucleotide polymorphisms. J. Stat. Softw..

[27] Baenziger PS (2004). Registration of ‘Goodstreak’ Wheat. Crop Sci..

[28] Ward, R. A. Identification of Stem Rust Resistance in Three Synthetic Wheat Populations. (Unversity of Nebraska-Lincoln, 2012).

[29] Jin Y, Singh RP (2006). Resistance in U.S. wheat to recent Eastern African isolates of Puccinia graminis f. sp. tritici with virulence to resistance gene Sr31. Plant Dis..

[30] Kolmer JA, Jin Y, Long DL (2007). Wheat leaf and stem rust in the United States. Aust. J. Agric. Res..

[31] Moreno-Sevilla B, Baenziger PS, Peterson CJ, Graybosch RA, Mcvey DV (1994). 1BL/1RS Translocation: Agronomic performance of F3-derived lines from a inter Wheat cross. Crop Sci..

[32] Crespo-Herrera LA, Garkava-Gustavsson L, Åhman I (2017). A systematic review of rye (Secale cereale L.) as a source of resistance to pathogens and pests in wheat (Triticum aestivum L.). Hereditas.

[33] Turner MK, Jin Y, Rouse MN, Anderson JA (2016). Stem rust resistance in ‘Jagger’ winter wheat. Crop Sci..

[34] McIntosh, R., Wellings, C. & Park, R. *Wheat Rusts*: *an Atlas of Resistance Genes*. *Plant Breeding* (1995).

[35] Tian-ya L, Xian-xin W, Xiao-feng X, Wan-lin W, Yuan-yin C (2016). Postulation of seedling stem rust resistance genes of Yunnan wheat cultivars in China. Plant Prot. Sci..

[36] Tsilo TJ, Jin Y, Anderson JA (2008). Diagnostic microsatellite markers for the detection of stem rust resistance gene Sr36 in diverse genetic backgrounds of wheat. Crop Sci..

[37] Ejaz M (2012). Genetic variation for markers linked to stem rust resistance genes in Pakistani wheat varieties. Crop Sci..

[38] Mago R (2005). Development of PCR markers for the selection of wheat stem rust resistance genes Sr24 and Sr26 in diverse wheat germplasm. Theor. Appl. Genet..

[39] Olson EL (2010). Genotyping of U.S. wheat germplasm for presence of stem rust resistance genes Sr24, Sr36 and Sr1RS Amigo. Crop Sci..

[40] Kassa MT (2016). Genetic mapping of SrCad and SNP marker development for marker-assisted selection of Ug99 stem rust resistance in wheat. Theor. Appl. Genet..

[41] Lopez-Vera EE (2014). Resistance to stem rust Ug99 in six bread wheat cultivars maps to chromosome 6DS. Theor. Appl. Genet..

[42] Mourad AMI (2018). Genetic architecture of common bunt resistance in winter wheat using genome- wide association study. BMC Plant Biol..

[43] Kumar J (2011). Advances in genomics resources for improving food legume crops. J. Agric. Sci..

[44] Ayliffe MA, Lagudah ES (2004). Molecular genetics of disease resistance in cereals. Ann. Bot..

[45] Hundertmark M, Hincha DK (2008). LEA (Late Embryogenesis Abundant) proteins and their encoding genes in Arabidopsis thaliana. BMC Genomics.

[46] Goff KE, Ramonell KM (2007). The role and regulation of receptor-like kinases in plant defense. Gene Regul. Syst. Biol..

