# Supplementary material for: Molecular marker dissection of stem rust resistance in Nebraska bread wheat germplasm
Source: Sci Rep. 2019 Aug 12;9:11694. doi: 10.1038/s41598-019-47986-9 (PMC6691005; doi:10.1038/s41598-019-47986-9)
Supplement: Supplementary file 1 — supplementary tables 1 (List of markers used to detect the presence of the different stem rust resistance genes, the expected band size, primer sequence and amplification conditions of each marker) and 2 (List of differential lines used as a positive control in marker-assisted selection to detect the right allele band size, the pedigree of each isoline and source as described on http://rusttracker.cimmyt.org/?page_id=30. ID was added to be used in the text.) [file 41598_2019_47986_MOESM1_ESM.docx]

**Molecular marker dissection of stem rust resistance in Nebraska bread wheat germplasm**

Amira Mourad ^(1),(4)^*, Ahmed Sallam ^(1),(5)^, Vikas Belamkar ^(1)^, Stephen Wegulo ^(2)^, Guihua Bai^(3)^, Ezzat Mahdy ^(4)^, Bahy Bakheit ^(4)^, Atif Abo El-Wafa ^(4)^, Yue Jin ^(6)^ and P. Stephen Baenziger ^(1)^

^(1)^ Department of Agronomy and Horticulture, Plant Science Hall, UNL, USA. ^(2)^ Department of Plant Pathology - Plant Science Hall, UNL, USA. ^(3)^ USDA-ARS Hard Winter Wheat Genetics Research Unit, 4008 Throckmorton Hall, Manhattan, KS, USA, ^(4)^ Agronomy Department, Faculty of Agriculture, Assiut University, Egypt. ^(5)^ Department of Genetics, Faculty of Agriculture, Assiut University, Egypt. ^(6)^ USDA-ARS Cereal Disease Lab, St. Paul, MN.

***Corresponding author**:

Amira Mourad

Address: Department of Agronomy, Faculty of Agriculture, Assiut University, Asyut, Egypt

Email: amira_mourad@aun.edu.eg

ORCID : 0000-0001-6879-2559

Supplementary Table 1. List of markers used to detect the presence of the different stem rust resistance genes, the expected band size, primer sequence and amplification conditions of each marker.

| **Gene** | **marker** | **Marker type** | **Expected band size** | **Sequence** | **Amplification conditions** |
| --- | --- | --- | --- | --- | --- |
| *SrTmp* | Xbarc183 | SSR | 169 | F: 5'CCCGGGACCACCAGTAAGT 3'  R: 5'GGATGGGGAATTGGAGATACAGAG3' | 5 minutes at 94 degrees; 35 cycles (94 degrees for 30 sec; 58 degrees for30 sec; 72 degrees for 30sec) 10 minutes at 72 c |
|  | barc20 | SSR | -- | F: 5'GCGATCCACACTTTGCCTCTTTTACA3'  R: 5'GCGATGTCGGTTTTCAGCCTTTT 3' | 5 minutes at 94 degrees; 35 cycles (94 degrees for 30 sec; 53 degrees for30 sec; 72 degrees for3 30sec) 10 minutes at 72 c |
|  | gpw5182 | SSR | 174 | F: 5'TCCACTTCACTAACAAACACGG3'  R: 5'AAAAGCTGTATAGGCAGTTCGC3' | 5 minutes at 94 degrees; 40 cycles (94 degrees for 1 min; 60 degrees for 1 min ; 72 degrees for 2 min); 10 minutes at 72 c |
| *Sr36* | wmc477 | SSR | 167 or 190 | F: 5'CGTCGAAAACCGTACACTCTCC3'  R: 5'GCGAAACAGAATAGCCCTGATG3' | 5 min at 94 degrees C; 40 cycles with 1 min 94 degree C, 1 min at 61 degree C, and 2 min at 72 degree C; and a final extension step of 10 min at 72 degree C |
|  | Xstm773-2 | SSR | 155 | F: 5'ATG GTT TGTTGTGTTGTGTGTAGG3'  R: 5'AAACGCCCCAACCACCTCTCTC3' | 94°C, 10 min, (7 cycles, 92°C, 1 min 64°C, 1 min 72°C, 1 min) , (5 cycles 92°C, 1 min 57°C, 1 min 72°C, 1 min), (10-25 cycles 92°C, 30 s 55°C, 1 min 72°C, 1 min) 72°C, 10 min |
|  | Xgwm319 | SSR | 170 | F: 5' GGTTGCTGTACAAGTGTTCACG3'  R: 5'CGGGTGCTGTGTGTAATGAC3' | 3 min at 94 degree C; 45 cycles with 1 min at 94 degree C, 1 min at 55 degree C, and 2 min at 72 degree C; and a final extension step of 10 min at 72 degree C |
|  | gwm271 | SSR | 171 or195 | F: 5'CAAGATCGTGGAGCCAGC3'  R: 5'AGCTGCTAGCTTTTGGGACA3' | 3 minutes at 94 degrees; 40 cycles (94 degrees for 1 min; 60 degrees for 1 min ; 72 degrees for 2min); 10 minutes at 72 c |

Supplementary Table 2. List of differential lines used as a positive control in marker assisted selection to detect the right allele band size, the pedigree of each isoline and source as described on <http://rusttracker.cimmyt.org/?page_id=30>. ID was added to be used in the text.

| **Gene** | **Differential line** | **Origin/pedigree** | **Source** | **ID** |
| --- | --- | --- | --- | --- |
| *Sr36* | W2691SrTt-1 CI 17385 | CI 12632 T. timopheevii | Jin, USDA | ISr36 |
| *Sr38* | Trident | Spear*4/VPM (PI 519303) | Park, Australia | ISr38 |
| *Sr31* | Kavkaz/Federation4 | Kavkaz | Pretorius, SA | ISr31 |
| *Sr1RS^Amigo^* | Amigo |  | Jin, USDA | ISrAmigo |
| *Sr24* | LcSr24Ag | Little Club/Agent (CI 13523) | Jin, USDA | ISr24 |
| *SrTmp* | CnsSrTmp | Triumph 64 (CI 13679)/Chinese Spring | Jin, USDA | ISrTmp |
